# Increased Risk of Neurodegenerative Dementia after Benign Paroxysmal Positional Vertigo

**DOI:** 10.3390/ijerph181910553

**Published:** 2021-10-08

**Authors:** So Young Kim, Dae Myoung Yoo, Chanyang Min, Hyo Geun Choi

**Affiliations:** 1 Department of Otorhinolaryngology-Head & Neck Surgery, CHA Bundang Medical Center, CHA University, Seongnam 13496, Korea; sossi81@hanmail.net; 2 Hallym Data Science Laboratory, Hallym University, College of Medicine, Anyang 14068, Korea; ydm1285@naver.com (D.M.Y.); joicemin@naver.com (C.M.); 3 Graduate School of Public Health, Seoul National University, Seoul 08826, Korea; 4 Department of Otorhinolaryngology-Head & Neck Surgery, Hallym University College of Medicine, Anyang 14068, Korea

**Keywords:** benign paroxysmal positional vertigo, dementia, risk factors, cohort studies, epidemiology

## Abstract

The aim of the present study was to estimate the risk of dementia in patients with benign paroxysmal positional vertigo (BPPV), using a population cohort. Data from the Korean National Health Insurance Service-National Sample Cohort for the population ≥60 years of age from 2002 to 2013 were collected. A total of 11,432 individuals with dementia were matched for age, sex, income, region of residence, hypertension, diabetes, and dyslipidemia with 45,728 individuals comprising the control group. The crude (simple) and adjusted odds ratios (ORs) of dementia in BPPV patients were analyzed using non-conditional logistic regression analyses. Subgroup analyses were conducted according to age and sex. A history of BPPV characterized 5.3% (609/11,432) of the dementia group and 2.6% (1,194/45,728) of the control group (*p* < 0.001). The adjusted OR of dementia for BPPV was 1.14 (95% CI = 1.03–1.26, *p* = 0.009). In subgroup analyses according to age and sex, males had higher ORs of dementia for BPPV. BPPV increases the risk of dementia in the 60 years of age or older population.

## 1. Introduction

A cross-sectional study reported that patients with cognitive impairment, such as mild cognitive impairment and Alzheimer’s dementia, show a high risk of vestibular dysfunction [1]. The vestibular system is known to affect cognitive functions, such as visuospatial ability, memory, and attention [2,3]. In a cross-sectional study that included 308 adults with neurodegenerative disorders, the authors observed that attentional and visuospatial cognitive abilities were correlated with increased dizziness [2]. Additionally, the prevalence of hippocampal atrophy was higher in patients with chronic bilateral vestibular dysfunction than in controls [4]. The authors hypothesized that optimal vestibular function is essential to maintain the phylogenetically ancient hippocampal function, such as spatial aspects of memory processing for navigation [5]. At the molecular level, unilateral vestibulopathy was associated with decreased hippocampal expression of N-methyl-D-aspartate receptors [4]. Therefore, it is reasonable to conclude that vestibular disorders may be associated with dementia.

Benign paroxysmal positional vertigo (BPPV) is the most common cause of vestibular vertigo in elderly individuals [6]; approximately 30% of individuals aged ≥70 years report an episode of BPPV at least once in their lifetime [7,8]. Moreover, the response to canalith repositioning therapy tends to be lower and the rate of recurrent BPPV is higher in elderly patients than in younger individuals [6,9,10]. BPPV shows a benign course; therefore, its association with other diseases is often ignored. A recent study reported BPPV as a predictor of dementia [11].

Both direct and indirect mechanisms may underlie the association between BPPV and dementia. With regard to a direct mechanism, an increased risk of falls in patients with BPPV may result in a high risk of head trauma and physical inactivity, which predispose these patients to dementia [1,12]. The sudden onset of vertigo observed in patients with BPPV reportedly increases the risk of falls in elderly patients [13]. With regard to indirect mechanisms, common cardiovascular or cerebrovascular pathophysiologies may mediate the onset of dementia in patients with BPPV. Recent studies have suggested a causal association between cardiovascular diseases, including hypertension and ischemic heart disease, and BPPV [14,15]. Vascular disorders, including hypertension, diabetes, and dyslipidemia, are known to increase the risk of dementia [16,17,18].

We hypothesized that BPPV could increase the risk of dementia. We searched the PubMed and Embase databases using the key words “BPPV” and “dementia” and included all entries until September 2021 in our search. We identified only one population cohort study that reported an increased risk of dementia in patients with BPPV [11]. However, the study did not include a control group matched for comorbidities. The characteristics of early-onset dementia differ from those of late-onset dementia; therefore, inclusion of individuals aged ≥20 years in a study with a follow-up of 10 years may negatively affect the association between dementia and BPPV [19]. In this study, we investigated the association between BPPV and dementia in an older Korean population extracted from a national sample cohort. This study extended previous research in the field; we investigated a history of BPPV as an independent risk factor in patients aged ≥60 years diagnosed with dementia.

## 2. Materials and Methods

### 2.1. Ethical Considerations

The Ethics Committee of Hallym University (2014-I148) approved the use of the data. The need for written informed consent was waived by the Institutional Review Board.

### 2.2. Study Population and Data Collection

This matched case–control study relied on data from the Korean National Health Insurance Service-National Sample Cohort (NHIS-NSC). The Korean NHIS selects samples directly from the database of the entire population to prevent non-sampling errors [20]. For this study, ~2% of the samples (one million) were selected from the entire Korean population (50 million). The selected data could be classified at 1476 levels (including age (18 categories), sex (2 categories), and income level (41 categories)) using randomized stratified systematic sampling methods via proportional allocation to represent the entire population. After data selection, the appropriateness of the sample was verified as described previously [21]. The details of the methods used to perform these procedures are provided by the National Health Insurance Sharing Service [22]. The cohort database used in our study included (i) personal information, (ii) health-insurance claim codes (procedures and prescriptions), (iii) diagnostic codes using the International Classification of Disease-10 (ICD-10), (iv) death records from the Korean National Statistical Office (using the Korean Standard Classification of disease), (v) socio-economic data (residence and income), and (vi) medical examination data for each participant during the period 2002–2013.

As all Korean citizens are assigned a 13-digit resident registration number that is retained from birth to death, exact population statistics can be determined from the resulting database. All Koreans must enroll in the NHIS. The 13-digit resident registration number is used by Korean hospitals and clinics to register individual patients in the medical insurance system. Therefore, the risk of overlapping medical records is minimal, even if a patient moves from one place to another. Moreover, all medical treatments in Korea can be tracked, without exception, using the HIRA (Health Insurance Review & Assessment Service) system. In Korea, a notice of death must be submitted to an administrative entity before a funeral can be held. The cause and date of death are recorded in the death certificate, which is prepared by a medical doctor.

### 2.3. Participant Selection

Among the 1,125,691 individuals with 114,369,638 medical claim codes, those diagnosed with dementia were included in the study. Dementia was defined based on a diagnosis of Alzheimer’s disease (G30) or dementia in Alzheimer’s disease (F00). To ensure the accuracy of the diagnosis, only those participants treated at least twice were included in the study. The reliability of the dementia diagnosis is described in the Supplemental Digital Content.

The study population consisted of 13,102 dementia patients diagnosed between 2002 and 2013. BPPV was diagnosed based on ICD-10 codes (H811) and at least two treatments for this condition between 2002 and 2013.

**Figure 1:**
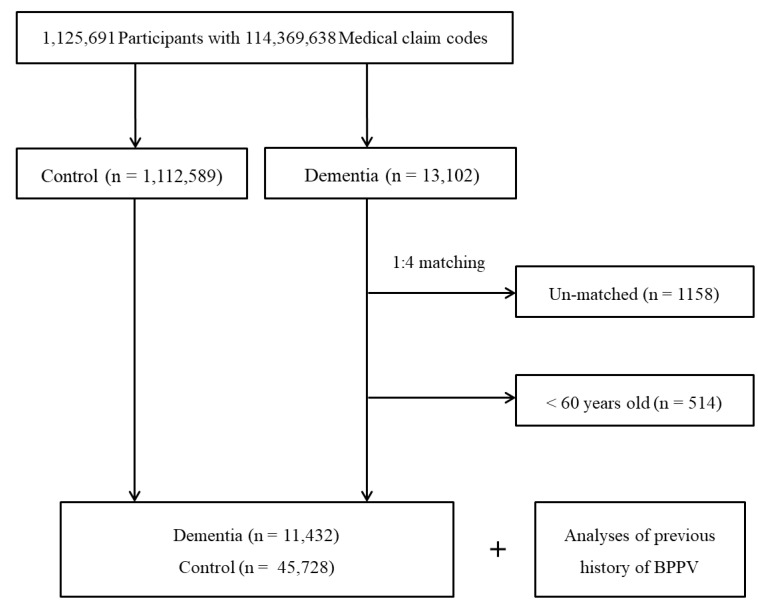
Schematic illustration of the participant-selection process used in the study. From a total of 1,125,691 participants, 11,432 patients with dementia were matched with 45,728 controls for age, sex, income, region of residence, and medical histories.

The dementia patients were matched 1:4 with members of a control group selected from the original population (n = 1,112,589) and consisting of individuals never diagnosed with dementia between 2002 and 2013. The matches were processed for age, sex, income, region of residence, and a medical history of hypertension, diabetes, or dyslipidemia. To prevent bias in the selection of matched participants, control-group participants were sorted by first assigning them a random number, which was then used in their selection, beginning with the highest and ending with the lowest number. It was assumed that the relation to the index date of the matched control participants was the same as that of each matched participant with dementia. Therefore, a person in the control group who died before the index date was excluded. Dementia participants for whom a sufficient number of matching control participants could not be identified were excluded (n = 1158), as were participants younger than 60 years of age who had been previously diagnosed with dementia (n = 514). In the young population, dementia is rare and often has a specific underlying etiology. Finally, using 1:4 matching, 11,432 dementia patients and 45,728 controls were included in the study (Figure 1). However, the two groups were not matched for ischemic heart disease and cerebral stroke history, as the stricter matching would have increased the exclusion of dementia patients due to a lack of controls. After matching, both groups were analyzed for a previous history of BPPV.

### 2.4. Variables

All variables including age, income level, region of residence, and comorbidities were based on the index date. Six age groups were defined according to 5-year intervals, ranging from 60–64 to 85+ years of age. Income groups were initially divided into 41 classes (1 health-insurance-assistance class, 20 self-employment health-insurance classes, and 20 employment health-insurance classes) but then re-categorized into five classes, ranging from lowest (class 1) to highest (class 5). Sixteen regions of residence were defined according to administrative district. These regions were regrouped as urban (Seoul, Busan, Daegu, Incheon, Gwangju, Daejeon, and Ulsan) and rural (Gyeonggi, Gangwon, Chungcheongbuk, Chungcheongnam, Jeollabuk, Jeollanam, Gyeongsangbuk, Gyeongsangnam, and Jeju).

The prior medical histories of the patients and controls were evaluated using ICD-10 codes. To ensure the accuracy of the diagnoses, hypertension (I10 and I15), diabetes (E10–E14), dyslipidemia (E78), and head trauma (S00–S09) were regarded as present if treated at least twice, and Meniere’s disease (H810), ischemic heart disease (I24 and I25), and cerebral stroke (I60–I63) strokes as present if treated at least once.

### 2.5. Statistical Analyses

Chi-square tests were used to compare the general characteristics of the dementia and control groups. The odds ratio (ORs) of BPPV for dementia was analyzed using unconditional logistic regression analysis together with crude (simple) and adjusted (ischemic heart disease, stroke, Meniere’s disease, and head-trauma histories) models and calculating 95% confidence intervals (CIs). Age, sex, income, region of residence, hypertension, diabetes mellitus, and dyslipidemia were stratified in a conditional logistic regression model. For the subgroup analyses, participants were divided with respect to age (<75 years old, ≥75 years), sex, income, region of residence, hypertension, diabetes, dyslipidemia, ischemic heart disease, stroke, Meniere’s disease, and head trauma. Two-tailed analyses were conducted. A *p* value < 0.05 was considered to indicate statistical significance. The analyses were conducted using SPSS v. 22.0 (IBM, Armonk, NY, USA).

## 3. Results

A history of BPPV was documented in 5.3% (609/11,432) of the dementia patients vs. 2.6% of the controls (1194/45,728; *p* < 0.001, Table 1). The mean follow-up duration from BPPV to index date was 49.97 (standard deviation [SD] = 35.97) for the dementia group and 50.34 (SD = 33.90) for the control group. Age, sex, income, region of residence, and history of hypertension, diabetes, and dyslipidemia were matched between the dementia and control groups. A history of ischemic heart disease, cerebral stroke, Meniere’s disease, and head trauma were higher in the dementia group than in the control group.

The OR for BPPV was higher in the dementia group than in the control group (OR = 1.29, 95% CI = 1.18–1.42; *p* < 0.001, Table 2). The higher risk was maintained after adjusting for ischemic heart disease, cerebral stroke, Meniere’s disease, and head trauma (adjusted OR = 1.14, 95% CI = 1.03–1.26; *p* = 0.009).

In the subgroup analysis according to sex, the OR for BPPV was higher in males (adjusted OR = 1.32, 95% CI = 1.09–1.61, *p* = 0.005, Table 2). There was no increase in the OR for BPPV in female dementia patients.

According to income and region of residence, the subgroups of low income and rural residence showed high OR for BPPV in dementia patients (each of *p* < 0.05). According to the medical histories, the subgroups with histories of hypertension, diabetes, or dyslipidemia, and no histories of ischemic heart disease, stroke, Meniere’s disease, or head trauma demonstrated increase in the OR for BPPV in dementia patients (each of *p* < 0.05, Table 3).

## 4. Discussion

After adjustment for age, sex, income, region of residence, and medical history, the risk of dementia was higher in the BPPV than in the matched control group. Among patients with BPPV, male sex, high income, rural residence, a history of hypertension, diabetes, or dyslipidemia, as well as absence of a history of ischemic heart disease, stroke, Meniere’s disease, and head trauma were associated with a high risk of dementia. These findings extend those of previous studies to a larger population with a matched control group.

A previous population cohort study also reported an increased risk of dementia in patients with BPPV (a 1.24-fold higher hazard ratio of dementia in patients with BPPV, 95% confidence interval 1.09–1.40, *p* < 0.001) [11]. However, the control group in the study was matched only for age and sex. Although we adjusted for possible confounders, the effects of unmatched comorbidities on the association between independent and dependent variables cannot be completely excluded [23]. Furthermore, the study included individuals aged ≥20 years, whereas our study included only those aged ≥60 years because dementia is rare in young individuals and usually shows a specific etiology.

BPPV may represent an initial manifestation of degenerative nervous system changes. Vestibular and macular degeneration may lead to otoconial detachment; studies have reported neuronal degenerative changes of the saccular macula in patients with BPPV [24,25]. Degenerative changes in the utricular macula have also been observed in patients with BPPV, who underwent surgery for posterior semicircular canal occlusion [26]. A loss of ganglion cells in the superior or inferior vestibular nerve and saccular ganglion cells, involving approximately 50% and 30%, respectively, of the temporal bone, have been reported in patients with BPPV [27]. Neuronal degeneration is known to affect the phylogenetically older neurons in patients with dementia [28]. The vestibular system is the phylogenetically oldest sensory system [29]; therefore, its degeneration may represent an early or prodromal stage of dementia. Moreover, symptoms of spinning-type vertigo are more prominent than those associated with cognitive impairment; therefore, BPPV tends to be diagnosed earlier than full-blown dementia.

Complications of BPPV, including falls, inactivity, and reduced social activity may contribute to the risk of dementia in patients with BPPV. BPPV is an important contributor to falls in the elderly population [30]. In a retrospective study of elderly patients with BPPV, canalith repositioning therapy was shown to reduce the number of falls [13]. Falls are associated with dementia and serve as an initial presentation in approximately 30% of patients with dementia [31]. Falls may result in traumatic brain injuries, which are shown to be associated with dementia. However, in our subgroup analysis, patients with a history of head trauma did not show an association between BPPV and dementia. Therefore, additional contributors may mediate the association between BPPV and dementia. Comorbidities such as inactivity, anxiety, and depression tend to increase the risk of dementia [32]. Additionally, medications, such as benzodiazepines administered to reduce vertigo or anxiety in patients with BPPV may affect the development of dementia [33]. However, the effects of anxiolytics on subsequent dementia remain controversial [34]. A case–control study reported no definite association between benzodiazepine administration and subsequent dementia [34]. Further studies are warranted to investigate the effects of anxiolytics on dementia.

Shared pathophysiological mechanisms between BPPV and dementia may affect the risks associated with both conditions. A recent study observed that vestibular-system ischemia increased the risk of BPPV [15] and that patients with BPPV showed a high risk of cerebrovascular diseases, including stroke and migraine [15,35]. The association between BPPV and cardiovascular disorders was attributed to ischemia of the feeding arteries of the vestibular system [36]. Vascular compromise in hypertension and heart disease is shown to increase the risk of dementia [37]. In our subgroup analyses based on a history of comorbidities, we observed a definite association between BPPV and an increased risk of dementia in patients with hypertension, diabetes, and dyslipidemia. The high predisposition to both BPPV and dementia may contribute to the higher association between BPPV and dementia. Although we matched and adjusted for confounders, we cannot completely exclude the potential effects of these comorbidities on the association between BPPV and dementia.

Our study also highlighted the higher risk of dementia in men with BPPV. A previous study reported that education level, social activities, and alcohol consumption interact with sex to affect cognitive function [38] and may also play a role in the sex-based differences in the risk of dementia in patients with BPPV. Furthermore, in this study, high income and rural residence affected the association between BPPV and the increased risk of dementia. Access to healthcare is invariably affected by income levels. This study was based on health-claims data; therefore, it is reasonable to conclude that clinical visits for evaluation of BPPV may be higher in patients with a high income.

The present study was based on nationwide representative data, the validity of which was verified by a previous study [21]. The National Health Interview Survey data include all Korean citizens, without exception; therefore, there were no missing participants. The control group was randomly selected and matched for age, sex, income, region of residence, and a medical history of hypertension, diabetes, and dyslipidemia. We included income and region of residence because these factors tend to determine the availability of healthcare.

Following are the limitations of our study. BPPV was diagnosed by a physician in patients who were treated at least twice for the condition. However, BPPV was not categorized based on severity and subtypes. Various pathophysiological conditions, including cupulolithiasis, canal switch, and re-entry phenomenon are associated with BPPV [39]. The diagnosis of BPPV could be inaccurate, particularly in elderly patients owing to the vague history and difficulty in physical examination in this population [40]. Positional nystagmus of central origin may also manifest as BPPV [41]. Reportedly, approximately 97.5% of central positional nystagmus manifests as an atypical direction of nystagmus observed during the Dix–Hallpike maneuver; however, central positional nystagmus secondary to cerebellar or brainstem involvement may mimic and be misdiagnosed as BPPV [41]. The diagnosis of BPPV may be missed by general physicians; BPPV is more accurately diagnosed by otoneurologists than by general practitioners. Compared with healthy controls, patients with comorbidities are more likely to be diagnosed with BPPV owing to frequent clinical visits. Although we adjusted for several potential confounders, we did not consider a few others, including body mass index, smoking status, alcohol consumption, physical inactivity, social isolation, education level, and hearing loss. The dementia group in this study was selected based on the International Classification of Diseases, Tenth Revision, Clinical Modification codes and a history of treatment with more than one therapeutic intervention for the condition. The prevalence of dementia assumed in this study was comparable with that reported by the Central Dementia Center of Korea. However, data from the Health Insurance Review and Assessment Service National Patient Sample do not include details regarding the severity of dementia; additionally, we could not determine semicircular-canal involvement and eventual recovery from BPPV.

## 5. Conclusions

BPPV was related with an increased risk of dementia in patients ≥ 60 years of age. This association of BPPV with dementia was valid in patients with male sex, low income, rural residence, and medical histories of hypertension, diabetes, and dyslipidemia. The possible links of BPPV with dementia need to be considered when managing patients with BPPV.

## Figures and Tables

**Table 1:** General Characteristics of Participants.

Characteristics	Total Participants		
	Dementia (n, %)	Control Group (n, %)	Standardized Differences
Age (years old)			0.00
60–64	580 (5.1)	2320 (5.1)	
65–69	1289 (11.3)	5156 (11.3)	
70–74	2325 (20.3)	9300 (20.3)	
75–79	2978 (26.1)	11,912 (26.1)	
80–84	2705 (23.7)	10,820 (23.7)	
85+	1555 (13.6)	6220 (13.6)	
Sex			0.00
Male	3659 (32.0)	14,636 (32.0)	
Female	7773 (68.0)	31,092 (68.0)	
Income			
1 (lowest)	2858 (25.0)	11,432 (25.0)	0.00
2	1037 (9.1)	4148 (9.1)	
3	1371 (12.0)	5484 (12.0)	
4	1886 (16.5)	7544 (16.5)	
5 (highest)	4280 (37.4)	17,120 (37.4)	
Region of residence			0.00
Urban	4617 (40.4)	18,468 (40.4)	
Rural	6815 (59.6)	27,260 (59.6)	
Hypertension			0.00
Yes	8314 (72.7)	33,256 (72.7)	
No	3118 (27.3)	12,472 (27.3)	
Diabetes Mellitus			0.00
Yes	4060 (35.5)	16,240 (35.5)	
No	7372 (64.5)	29488 (64.5)	
Dyslipidemia			0.00
Yes	3558 (31.1)	14,232 (31.1)	
No	7874 (68.9)	31,496 (68.9)	
Ischemic heart disease			0.05
Yes	1703 (15.0)	5992 (13.1)	
No	9729 (85.1)	39,736 (86.9)	
Cerebral stroke			0.50
Yes	5524 (48.3)	11,475 (25.1)	
No	5908 (51.7)	34,253 (74.9)	
Meniere’s disease			0.03
Yes	651 (5.7)	2319 (5.1)	
No	10,781 (94.3)	43,409 (94.9)	
Head trauma			0.03
Yes	886 (7.8)	1887 (4.1)	
No	10,546 (92.3)	43,841 (95.9)	
BPPV (Benging Paroxysmal Positional Vertigo)			0.05
Yes	609 (5.3)	1914(2.6)	
No	10,823 (94.7)	43,814 (95.8)	

**Table 2:** Crude and adjusted odds ratios (95% confidence interval) of BPPV for dementia and in subgroup analysis according to age, sex, income, and region of residence.

Characteristics	No. of BPPV/	No. of BPPV/	ORs for Dementia				*p* for Interaction
	No. of Dementia (%)	No. of Control (%)	Crude†	*p*-Value	Adjusted †,‡	*p*-Value	
Total participants (n = 57,160)							
BPPV	609/11,432 (5.3)	1914/45,728 (4.2)	1.29 (1.18–1.42)	<0.001 *	1.14 (1.03–1.26)	0.009 *	
Age < 75 years old (n = 20,970)							0.245
BPPV	216/4194 (5.2)	624/16,776 (3.7)	1.41 (1.20–1.65)	<0.001 *	1.18 (0.99–1.39)	0.062	
Age ≥ 75 years old (n = 36,190)							
BPPV	393/7238 (5.4)	1290/28,952 (4.5)	1.23 (1.10–1.39)	<0.001 *	1.12 (0.99–1.26)	0.066	
Men (n = 18,295)							0.063
BPPV	158/3659 (4.3)	428/14,636 (2.9)	1.50 (1.25–1.81)	<0.001 *	1.32 (1.09–1.61)	0.005 *	
Women (n = 38,865)							
BPPV	451/7773 (5.8)	1486/31,092 (4.8)	1.23 (1.10–1.37)	<0.001 *	1.09 (0.97–1.22)	0.148	
Low income (n = 26,330)							0.651
BPPV	262/5266 (5.0)	795/21,064 (3.8)	1.34 (1.16–1.55)	<0.001 *	1.19 (1.02–1.38)	0.026 *	
High income (n = 30,830)							
BPPV	347/6166 (5.6)	1119/24,664 (4.5)	1.26 (1.11–1.42)	<0.001 *	1.10 (0.97–1.26)	0.134	
Urban (n = 23,085)							0.920
BPPV	242/4617 (5.2)	762/18,468 (4.1)	1.29 (1.11–1.50)	0.001 *	1.14 (0.98–1.33)	0.096	
Rural (n = 34,075)							
BPPV	367/6815 (5.4)	1152/27,260 (4.2)	1.29 (1.15–1.46)	<0.001 *	1.14 (1.00–1.29)	0.046 *	

* Odd ratios on unconditional logistic regression model, Significance at *p* < 0.05. † Age, sex, income, region of residence, hypertension, diabetes mellitus, and dyslipidemia were stratified in a conditional logistic regression model. ‡ Adjusted model was adjusted for ischemic heart disease, cerebral stroke, Meniere’s disease, and head trauma.

**Table 3:** Subgroup analysis of crude and adjusted odds ratios (95% confidence interval) of BPPV for dementia according to hypertension, diabetes mellitus, dyslipidemia, ischemic heart disease, cerebral stroke, Meniere’s disease, and head trauma.

Characteristics	No. of BPPV/	No. of BPPV/	ORs for Dementia				*p* for Interaction
	No. of Dementia (%)	No. of Control (%)	Crude †	*p*-Value	Adjusted †,‡	*p*-Value	
Non-hypertension (n = 15,590)							0.978
BPPV	112/3118 (3.6)	346/12,472 (2.8)	1.31 (1.05–1.63)	0.016	1.12 (0.89–1.41)	0.327	
Hypertension (n = 41,570)							
BPPV	497/8314 (6.0)	1568/33,256 (4.7)	1.29 (1.16–1.43)	<0.001 *	1.14 (1.03–1.27)	0.016 *	
Non-diabetes mellitus (n = 36,860)							0.099
BPPV	343/7372 (4.7)	1142/29,488 (3.9)	1.21 (1.07–1.37)	0.002 *	1.07 (0.94–1.22)	0.288	
Diabetes mellitus (n = 20,300)							
BPPV	266/4060 (6.6)	772/16,240 (4.8)	1.41 (1.22–1.63)	<0.001 *	1.24 (1.07–1.44)	0.005*	
Non-dyslipidemia (n = 39,370)							0.500
BPPV	323/7874 (4.1)	1051/31,496 (3.3)	1.24 (1.09–1.41)	0.001 *	1.11 (0.97–1.27)	0.125	
Dyslipidemia (n = 17,790)							
BPPV	286/3558 (8.0)	863/14,232 (6.1)	1.36 (1.18–1.56)	<0.001 *	1.17 (1.02–1.36)	0.031 *	
Non-ischemic heart disease (n = 49,465)							0.107
BPPV	500/9729 (5.1)	1546/39,736 (3.9)	1.34 (1.21–1.48)	<0.001 *	1.18 (1.06–1.31)	0.003*	
Ischemic heart disease (n = 7695)							
BPPV	109/1703 (6.4)	368/5992 (6.1)	1.05 (0.84–1.30)	0.693	0.97 (0.77–1.22)	0.781	
Non-cerebral stroke (n = 40,161)							<0.001*
BPPV	287/5908 (4.9)	1156/34,253 (3.4)	1.46 (1.28–1.67)	<0.001 *	1.46 (1.27–1.67)	<0.001 *	
Cerebral stroke (n = 16,999)							
BPPV	322/5524 (5.8)	758/11,475 (6.6)	0.88 (0.77–1.00)	0.052	0.90 (0.79–1.04)	0.146	
Non-Meniere’s disease (n = 54,190)							0.934
BPPV	496/10,781 (4.6)	1564/43,409 (3.6)	1.29 (1.16–1.43)	<0.001 *	1.14 (1.02–1.26)	0.020 *	
Meniere’s disease (n = 2970)							
BPPV	113/651 (17.4)	350/2319 (15.1)	1.18 (0.94–1.49)	0.160	1.13 (0.89–1.43)	0.318	
Non-head trauma (n = 54,387)							0.163
BPPV	560/10,546 (5.3)	1804/43,841 (4.1)	1.31 (1.19–1.44)	<0.001 *	1.16 (1.05–1.28)	0.005 *	
Head trauma (n = 2773)							
BPPV	49/886 (5.5)	110/1887 (5.8)	0.95 (0.67–1.34)	0.754	0.90 (0.63–1.29)	0.571	

* Odds ratios from a conditional logistic regression model in subgroups according to hypertension, diabetes mellitus, and dyslipidemia, and odds ratios from a unconditional logistic regression model in subgroups according to ischemic heart disease, cerebral stroke, Meniere’s disease, and head trauma. Significance at *p* < 0.05, † Age, sex, income, region of residence, hypertension, diabetes mellitus, and dyslipidemia were stratified in a conditional logistic regression model ‡ Adjusted model was adjusted for ischemic heart disease, cerebral stroke, Meniere’s disease, and head trauma in a conditional logistic regression model, and age, sex, income, region of residence, hypertension, diabetes mellitus, dyslipidemia, ischemic heart disease, cerebral stroke, Meniere’s disease, and head trauma in an unconditional logistic regression model.

## Data Availability

Releasing of the data by the researcher is not legally permitted. All data are available from the database of the Korea Center for Disease Control and Prevention. The Korea Center for Disease Control and Prevention allows data access, at a particular cost, for any researcher who promises to follow the research ethics. The data of this article can be downloaded from the website after agreeing to follow the research ethics.
